# Supplementary material for: Are carbon emissions trading and green financial instruments synergistic? -Comprehensive quantitative research based on content analysis
Source: PLoS One. 2024 Mar 7;19(3):e0298601. doi: 10.1371/journal.pone.0298601 (PMC10919601; doi:10.1371/journal.pone.0298601)
Supplement: S1 Data — (ZIP) [file pone.0298601.s001.zip › Data/地级市Z_F工作报告.zip!/╔╜╬≈/╜·╓╨╩╨/└┤╫╘╣1⁄2╓┌║┼í╛┬φ┐╦╩2╛▌═°í┐_2014.docx]

各位代表:

　　现在,我代表市人民政府向大会报告政府工作,请予审议,并请市政协委员和其他列席人员提出意见。

　　一、2013年工作回顾

　　过去的一年,面对经济下行压力,全市上下认真贯彻落实中央、省一系列决策部署, 把握稳中求进总基调,抢抓综改试验和同城化发展机遇,凝心聚力谋发展、稳增长、促转型、惠民生,全市经济和社会发展稳中有为,稳中有进,稳中提效。

　　一是经济发展取得新成绩。地区生产总值增长9.1%;规模以上工业增加值增长13%;固定资产投资增长27.1%;社会消费品零售总额增长13.8%;公共财政预算收入增长16%;城镇居民人均可支配收入增长9.9%;农村居民人均纯收入增长13.3%。多项指标增幅高于全省平均水平,位次较上年前移。

　　二是产业转型迈出新步伐。深入开展“项目推进年”活动,全市项目储备、签约、落地、开工、建设、投产均超额完成省定任务;非煤产业工业增加值比重提高0.7个百分点,新兴产业投资增长48.6%,对全市产业转型升级形成有力的支撑和拉动。传统产业改造步伐加快。全市煤炭行业新增25个现代化矿井,新增产能1950万吨;焦化行业兼并重组取得实质性进展,企业数量由27户减少至12户。新兴产业培育力度加大。以青云直升机、吉利汽车、平遥煤化光学新材料等项目为标志的装备制造业、新材料工业发展提档加速;以太铁货运物流中心为标志的现代物流业加快成长;旅游总收入突破300亿元,增长39.4 %,绵山获批5A景区,《又见平遥》效应凸显,晋中市通过国家旅游服务业标准化示范城市验收;文化产业增加值占GDP比重5.49%,继续保持全省前列。特色现代农业势头良好。粮食总产18.2亿公斤,再创历史新高;11个“一村一品”精品示范片区初步形成,新发展特色专业村243个,主导产业人均纯收入占到农民人均纯收入72%;新发展设施蔬菜6.1万亩、养殖(园区)237个、干果经济林20.2万亩;全市农产品加工企业销售收入135亿元;新发展农民合作社1256家,保持全省领先,培育生态庄园、沟域经济、家庭农场等经营大户140个;流转土地113万亩,占家庭承包耕地27.2%。园区承载能力提升。全市8个重点工业园区累计入驻企业516户,销售收入占全市40%,介休义安循环经济园区、灵石中煤循环经济园区、榆次工业园区收入均超过百亿元。

　　三是城乡面貌发生新变化。全市实施城镇化项目466项,完成投资266.5亿元。城镇化率提高1.5个百分点。加快太原晋中同城化发展。城区市政重点工程完成投资119.6亿元,供水、供热、供气、污水和垃圾处理率进一步提高,路网建设、城市扩容、环境改善、便民设施取得新进展,高校新区6.7万师生入住,科技创新城建设启动,完成汇通北路、迎宾街东延、文津街、乌金路等12条城市主要道路建设和晋中北高速出口扩容改造,大西铁路专线晋中站基本建成,城市轨道交通项目成功启动。推进特色城镇建设。10个县市完成大县城建设投资129.4亿元,村镇建设投资173.5亿元。阳黎高速昔左段即将通车,基本实现县县通高速。恋思、石膏山、泽城西安3个新水源工程下闸蓄水;东山供水、中部引黄晋中供水工程进展顺利。12个电力设施项目建成,新增变电容量403万千伏安。狠抓生态环境保护。约束性指标全部完成,PM2.5有效监测天数达标率超省定任务28.4个百分点;完成造林合格面积52万亩,林木绿化率增加1.2个百分点,榆次区通过省级林业生态县验收;城乡面貌明显改观,左权县、平遥县分别被命名为全国和全省文明县城,灵石、和顺分别被命名为国家园林县城和省级园林县城,灵石、左权成为全市首批国家卫生县城。

　　四是综改试验实现新突破。“108国道综合发展廊带”的项目建设、城市管理、土地流转、审批改革等取得实质性进展。积极建设区域资本市场,在全省率先挂牌运营晋中民间融资登记服务中心,天津股权交易所山西分市场不日挂牌运行,灵石县设立全国首支县域经济发展和城镇化建设私募基金。着力破解用地难题,获取用地周转指标近1.1万亩,露天采矿用地改革批准试点面积近4万亩,左权县在全省首家推出“土地银行”,引导民间资本实施土地开发。创新环境容量市场化交易,环保排污权交易开始网络受理业务。

　　五是民生事业得到新改善。全市财政用于民生领域支出增加31亿元,增长21.65%。建成保障性住房26832套,名列全省前茅。农村“五件实事”完成年度任务。城镇新增就业4.8万人,登记失业率1.95%,低于控制目标。居民消费价格涨幅3%,控制在年度目标以内。各类保险参保人数、保费征缴额进一步提高,城乡低保实现应保尽保。“爱心煤”政策惠及89万户农民家庭。第一轮学前教育三年行动计划全面完成,新改扩建公办标准化幼儿园175所,入园率高出全省14.1个百分点;义务教育均衡发展晋中模式在全国推广。县域医药卫生一体化综合改革和公立医院试点改革积极推进,新农合参合率达到99.37%。文化体制改革、文化惠民工程、文艺创作以及文物和非物质文化遗产保护传承取得新进展。全民健身活动蓬勃开展,竞技体育水平进一步提高。扶贫开发“双百工程”扎实推进,全市又有3.2万人脱贫。安全生产较大以上事故的起数和死亡人数均实现“双下降”,全年无重大事故。食品药品安全保障水平进一步提升。

　　六是政府作风焕发新气象。认真落实中央“八项规定”和省、市实施办法,以反对“四风”、服务群众和建设效能政府为重点,加强政府系统廉政建设和作风建设,政府公信力、执行力进一步提升。深入开展行政不作为专项整治,对10起典型事例进行了处理。引深行政审批制度改革,市级取消、下放审批和收费事项44项,“两集中两到位”改革取得突破。进一步理顺部门职能,改组市公投公司,优化政府投资项目代建机制。市直机关归并整合37个单位办公场所,清理清退超标办公用房14.28万平方米。市本级预算单位“三公”经费和会议费支出下降18.69%。

　　七是社会和谐呈现新局面。进一步加强民主法制建设,自觉接受人大法律监督、政协民主监督和社会舆论监督,全年共办理人大代表意见建议211件、政协委员提案371件,办复率100%,满意率和基本满意率提高3个百分点。深入推进“六五”普法,“法治晋中”建设取得新成效。完善信访工作机制,群众利益诉求渠道更加畅通。加强和创新社会治理,完善“大调解”工作体系, 深入开展群众性精神文明创建活动,依法打击各类违法犯罪活动,社会保持和谐稳定。国防教育和双拥工作深入开展,人口计生、妇女儿童、残疾人、老龄事业和红十字会、慈善公益事业积极发展,民族、宗教、外事、侨务工作进一步加强,气象、地震、人防和档案、史志、科普等工作都取得新成绩。市政府向全市人民承诺的重点实事全部兑现。

　　成绩来之不易。这是市委正确领导的结果,是市人大和市政协监督支持的结果,是全市广大干部群众团结奋斗的结果。我代表市人民政府,向全市各条战线的广大干部群众,向大力支持政府工作的政协委员、各民主党派、工商联和人民团体, 向驻地部队广大指战员、武警官兵、公安干警和国家、省驻地单位,向所有关心支持晋中发展的社会各界人士,表示崇高的敬意和衷心的感谢!

　　回顾一年的实践,我们深切体会到,做好政府工作必须坚持问题导向,强化担当意识,问题之前不回避,困难面前不低头,做狮子型干部,不做鸵鸟型干部,对人民群众有利的事就要率先力行,对晋中发展有益的事就要迎难而上。必须坚持结果导向,强化责任意识,言必行,行必果,立足目标定规划、抓过程、查绩效、要结果,不达目的不罢休。必须坚持民生导向,强化服务意识,吃百姓饭,为人民干,不做官老爷,甘当服务员,想问题、作决策、办事情,始终把解民忧、谋福祉作为根本追求。必须坚持市场导向,强化自我革新意识,坚持法无授权不可为,最大限度地简政放权,放开市场这只“看不见的手”,用好政府这只“看得见的手”,激活发展动力和创新潜力。

　　我们清醒地看到,当前发展中依然存在一些突出矛盾和问题:主要是资源依赖、发展粗放,经济增长乏力,项目支撑不足,转型发展任务艰巨;城镇化发展相对滞后,城乡和区域发展不平衡,4个国家级、省级扶贫开发重点县,36.78万贫困人口尚未脱贫;社会事业发展不足,公共设施投入及事关群众生产生活的突出问题还没有很好解决,就业、社会保障工作压力较大,安全稳定形势与人民群众期望还有差距;政府部门职能转变滞后,效能不高,作风漂浮,思想保守,环境不优,行政不作为、慢作为、乱作为现象仍然存在,腐败问题还时有发生。这些问题事关全局,我们必须以寝食难安的责任感和壮士断腕的勇气,自我革命,善做善成,倾力解决。

　　二、2014年工作安排

　　2014年,经济长期向好的基本面没有变。十八届三中全会开启了全面深化改革的新征程,改革红利、发展动力、市场活力将得到进一步释放。国家对中西部发展和资源型城市转型的政策支持力度不断加大。综改试验和同城发展的促进效应逐步显现,高校新区、科技创新城建设积蓄的势能正在形成,我们面临着难得的发展机遇。但是,前进道路上面临诸多挑战,外部经济形势严峻,不稳定、不确定因素依然存在。我市传统支柱产业经营困难,下行压力仍然较大。我们必须增强机遇意识和忧患意识,坚定信心,攻坚克难,推动各项工作再上新台阶。

　　做好今年的政府工作,要贯彻落实好市委三届五次全会提出的工作总要求:“深入贯彻落实党的十八大、十八届三中全会和省委十届五次全会暨全省经济工作会议精神,围绕建设全省‘四化’率先发展区总目标,坚持以科学发展为主题,以转变发展方式为主线,以全面深化改革为动力,以项目建设为抓手,以优化发展环境为保障,加强经济、政治、文化、社会、生态文明建设,以党的群众路线教育实践活动为抓手全面加强党的建设,为加快转型跨越发展、全面建成小康社会努力奋斗。”

　　主要预期指标是:地区生产总值增长9%,规模以上工业增加值增长13%,全社会固定资产投资增长25%,公共财政预算收入增长9%,社会消费品零售总额增长14%,城镇居民人均可支配收入增长10%,农民人均纯收入增长13%,城镇新增就业人数3.87万人,城镇登记失业率控制在4.2%以内,居民消费价格涨幅控制在3.5%左右。

　　约束性指标是:万元GDP综合能耗下降3.5%,万元GDP二氧化碳排放量下降3.7%,二氧化硫、化学需氧量、氨氮、氮氧化物排放量分别下降3.7%、4.5%、2.8%、9.1%,烟尘、粉尘排放量分别下降0.1%和0.2%,万元工业增加值用水量下降5%。

围绕上述目标,要重点抓好以下八方面的工作:

　　(一)坚持改革统领,突出综改攻坚,努力在重点领域和关键环节上取得突破。

　　把改革作为今年政府工作的首要任务,全面落实我市深化改革50条实施意见,积极开展“转型综改攻坚年”活动,扎实抓好12项重大事项、12项重大改革和30个重大项目,努力建设转型综改“先行区”。打造108廊带科学发展“升级版”。坚持市级统筹、规划引领、突出重点、分类推进,按照三大板块功能,建设“祁太平”现代农业示范区和国家旅游目的地,建设市城区高端装备制造业和现代物流基地,建设介休、灵石循环经济和新材料产业基地,打造综改集中连片试验区。坚持简政放权,扩大试点成果,统筹“一区两翼”产业和项目布局,科学制定统计、考评和利益分享机制,发展“飞地经济”。建立城镇组团私募股权基金,破解基础设施建设资金难题。实行差别化考评机制,引导各县(区市)按照版块功能定位推进重点项目和重大改革。

　　加快金融创新发展。激励间接融资,强化信用体系建设,完善考核激励机制,扎实开展中小企业规范化管理升级行动,进一步提高金融机构存贷比例;发展直接融资,挂牌启动天交所山西分市场,建立服务全省、辐射周边的中小企业股权交易市场,推动企业进行股份制改造和股权融资;规范民间融资,鼓励民间资本发展小额贷款、融资担保和私募股权基金,参股村镇银行和农信社改制;切实防范金融风险,优化金融生态。

　　推进土地管理制度和资源性产品价格改革。深化城乡建设用地增减挂钩改革,打破增减挂钩周转指标县域界面,指标在全市范围内统筹使用;探索建立土地开发多元投入机制和补偿奖励机制,鼓励民间资本参与土地开发;实行土地三维管理,有效利用浅层地下空间资源;建立节约集约用地考评体系,开展批而未供、未用和低效利用土地专项整治;试点开展城乡建设用地同等入市、同权同价改革和农村土地承包经营权确权登记,建立统一的城乡建设用地有形交易市场。深化资源性产品价格改革,完善居民水、电、气阶梯价格制度,推进供热分户计量改革。

　　深化财政体制和国有企业改革。进一步完善市县(区市)财政分配体制,明确支出责任,匹配事权财权;发挥财政资金的杠杆作用,创新投融资方式,优化政府投融资体系;改进预算管理,实行预决算和财务公开,强化预算约束,完善预算绩效评价体系,对国有资产经营和政府投资质量试行第三方专业机构诊断评审考核,提高财政资金使用效益。深化国有企业混合所有制改革,做好晋华、锦纶、轻机厂、格芙兰等企业改革后续工作,推进市药材公司、建设集团和厂办大集体改革,完成保安公司和武装押运公司改制任务。

　　统筹推进其它各项改革。深化投资体制改革,鼓励民营资本进入城市基础设施、文化、教育、卫生等行业领域。深化集体林权制度改革,开展林权流转和政策性森林保险工作,完善和启动县级生态公益林森林生态效益补偿制度。深化工商登记制度改革,推行认缴登记制,积极推进先照后证。加快推进政府机构改革、事业单位分类改革、卫生计生机构改革等工作。

　　(二)坚持项目支撑,突出落地投产,努力在增强发展后劲上取得突破。

　　以项目建设引领结构升级、产业转型升级,按照省委、省政府“项目见效年”的部署要求,坚持“5年5千亿投资”不动摇,狠抓项目储备、签约、落地、开工、建设和投产,确保今年全社会固定资产投资完成1180亿元以上。加大招商力度。充分发挥企业招商引资主体和开发区、产业园区招商引资排头兵作用,搭建项目寻源平台,集聚要素优势,扩大对外开放。以优势资源换项目、换投资、引技术、引人才,瞄准优势品牌企业和先进发达地区,强化互动交流,拓展合作领域,引进大项目好项目、新业态新技术,为转型升级积蓄后劲、提供支撑。有效对接相关重大招商活动,落实招商任务,全年招商引资1600亿元以上。

　　强化项目服务。树立服务项目就是服务发展,支持“项目见效”就是支持经济转型的理念,建立重点项目服务责任制,坚持领导联系重点工程、24小时直通车、目标责任管理等制度。重点对前三年的所有项目进行梳理剖析、分类指导、结对服务,创新方法,帮助企业和项目单位解决土地、资金等要素方面的实际问题,推动落地项目加快竣工、建成项目早日达效,确保全年完成项目投产1039亿元。

　　创新推进机制。建立项目寻源、评审推进机制,实行重大项目落地联席审议。强化项目建设过程管控和后评估,优惠及罚则与形象进度挂钩考核。建立重点项目服务团队,聘用社会专业人员,整合招商引资和项目建设工作力量,优化项目管理服务。设立新兴产业和传统产业项目专项资金各1亿元,市县财政1:1配套,进行以奖代补、贷款贴息和绩效奖励。实行重点工程分级管理制度,探索建立项目收益由引资相关方分享的激励机制。

　　(三)坚持结构调整,突出创新驱动,努力在加快产业转型上取得突破。

　　大力实施创新驱动发展战略,按照以煤为基、多元发展的要求,加快推进产业结构高级化、区域布局合理化、发展模式集聚化。做强做优传统产业。高标准推进现代化矿井建设,落实煤电协调发展机制,创新东山地区能源输出区域合作机制,建设东山县域煤炭就地清洁转化示范基地。大力发展煤层气地面抽采、提纯液化、管网建设等关联产业。推进煤电一体化发展,高效实施介休、灵石、寿阳三个低热值煤发电项目。推进焦化行业循环发展,积极实施化产延伸项目,加快建设介休和灵石焦化产业集聚区。完成红海玻璃文化艺术园、山西铸造产业研发展示中心、恒达精密铸造、伊利液态奶等项目建设,推进玻璃器皿、精密铸造、食品医药等特色产业兼并重组、集群发展,提高产业集中度和综合竞争力。

　　培育壮大新兴产业。大力发展现代装备制造业,加快全省新能源汽车城建设,推进北达新能源汽车及发动机、中航美运新能源特种车等项目全面开工,力争吉利汽车年底新车下线、引进纯电动汽车项目早日签约落地;支持装备制造业龙头企业开发新型高端产品,提高中小企业发展配套能力;积极扶持青云直升机项目建设,形成300架生产能力。培育发展新材料、生物医药、信息技术、北斗应用等高新产业。

　　加快发展现代服务业。打造文化“大晋中”。推进文化生态保护区建设,加快编制文化产业发展规划和城区文化产业创意园策划,实施晋华工业遗产保护开发、国际柔力球交流中心等项目,办好平遥国际摄影大展等文化活动,全市文化产业增加值增长20%以上。建设旅游“目的地”。探索成立旅游产业投资开发公司,试行集团化投资、运营和管理模式,整合重点旅游资源,打造古城大院板块、古镇名山板块、都市休闲板块和红色教育板块。巩固国家旅游服务业标准化示范城市创建成果,抓好平遥古城墙整体保护修缮开发、平遥古城生态文化旅游产业园、NAPA橡树谷、云竹湖休闲旅游度假区等项目,全市旅游总收入力争突破360亿元。推进现代物流、电子商务、研发设计等新兴服务业发展,加快建设太铁货运中心、苏宁电器配送中心等项目,扶持发展一批大型流通企业,加快培育1—2个销售10亿元、5—10个销售上亿元的现代流通企业集团,有效推进沃尔玛、王府井、汇通大厦等商业综合体建设。

　　构建区域创新体系。加强政校企对接,建立政产学研用一体化合作机制,搭建科研技术交易平台和服务平台。鼓励高校院所科研人员到企业兼职或领办、创办科技型企业,建设高校学生创新创业基地和科技企业孵化器。支持企业建立院士工作站、博士后流动站、技术研发中心,建设液压、纺机和玻璃器皿三个国家级产品质量监督检验中心。加快“智慧晋中”和“宽带晋中”建设。创新园区管理体制,支持晋中经济技术开发区、榆次工业园、祁县经济开发区扩区增容、一区多园发展,支持符合条件的工业园区升级提质,推进祁县开发区省级创新体制机制试点工作,增强园区吸引和接纳国内外大集团、大企业的能力。扶持中小微企业特别是科技型、创新型企业健康发展,推进中小企业创业基地建设。

　　(四)坚持“三农”为重,突出农民增收,努力在提升现代农业发展水平上取得突破。

　　始终把“三农”工作作为重中之重,以促进农民增收为核心,加快推进农业现代化,千方百计让农业强起来、农村美起来、农民富起来。大力发展特色现代农业。加快推进山西省农产品加工中心城市建设,做优“一村一品”特色,重点打造优势农产品集中片区,新发展专业村100个、专业乡镇10个,新发展设施蔬菜6万亩;抓好农产品流通和农村市场体系综合示范区国家级项目建设,完成7个农产品物流配送中心,4个乡镇商贸中心;扶持引导市级农产品加工龙头企业兼并重组,做大规模,做强品牌;加快搭建农产品网上交易平台,与淘宝网合作开设的淘宝晋中馆尽早开通交易,全市农产品加工企业销售收入达到140亿元以上。与山西农大合作建设农业科技创新园区,加快现代农业示范区上档升级。确保新组建的晋谷现代农业投资开发有限公司健康运营,集团化发展现代种养、技术贸易、农产品加工、物流配送等农业产业链,为全市现代农业建设和工程技术输出示范引路,促进农业科技成果产业化、市场化。推进太谷县国家级农业改革与建设试点工作,抓好农业综合开发、高效设施蔬菜基地及育苗中心、现代化规模养殖等项目,鼓励支持推广现代农业适用技术,打造全省特色现代农业示范区。创新农业经营机制,大力支持发展多种形式的新型农民合作组织,规范建设30个省级、50个市级、80个县级农民专业合作示范社,培育家庭示范农场30个。完成机械化深松整地20万亩。加大农民增收明白卡推广力度,切实提高农民收入。完成农户科学储粮罐4500套。

　　着力改善农村人居环境。巩固完善两轮“五个全覆盖”成果,全面完成省政府确定的“五件实事”任务,大力实施以农村基础设施和公共服务为重点的完善提质工程,以采煤沉陷治理、易地搬迁、危房改造为重点的农民安居工程,以垃圾污水治理为重点的城乡清洁工程,以美丽乡村建设为重点的宜居示范工程,尽快改善农村生产生活条件。

　　深入实施产业扶贫开发“双百”工程。以连片贫困区64个乡镇、1032个村为主战场,坚持产业扶贫开发、易地扶贫搬迁和劳动力素质提升“三位一体”工作路径,建立完善示范带动、项目支撑、政策支持、考核激励、精准扶贫等机制,扎实开展干部包村增收、机关定点扶贫和城乡“双联共建”活动,加大农企对接力度,力争今年再有3万以上贫困人口脱贫。

　　(五)坚持民生导向,突出城乡统筹,努力在推进特色新型城镇化建设上取得突破。

　　把城镇化作为最大的惠民工程,加快建设功能完善、特色彰显、环境优美、舒适宜居的美丽晋中,全市城镇化率力争提高2个百分点。加快同城发展。坚持新区集中连片大开发、旧区改造提质大突破思路,持续推进市城区扩容提质,重点实施8大类85项市政重点工程,力争今年完成120-140亿元投资。重点推进市博物馆、图书馆、科技馆、汇通大厦等项目建设,打造公共文化服务区、都市休闲生活区;加快大西铁路晋中沿线各站场建设和周边开发,建设人流物流集散基地;开工建设晋中第一人民医院和山大二院南院;抓好羊毫街、迎宾广场、晋华片区、迎宾东区4大片区,以及20个城中村、5个城市棚户区改造;完成锦纶路快速化改造、中都路北延等路网建设工程,改造工农巷等14条小街巷;实施3.5万户天然气置换和200万平方米热电联供工程;完成汇通路等5条城市主干街区、无照明设施或照明度低的15条街巷及高校新校区三座景观桥美化亮化工程,百草坡城郊森林公园建成开园,晋商公园三期、潇河公园二期、龙湖公园、社火公园建设基本完工;加快北部新城和高校新区文化娱乐中心、购物广场和创业就业中心等配套服务设施建设,做好山西科技创新城前期工作,启动核心区新城建设,同步推进晋中副中心建设,力争晋中市城区轨道交通工程具备开工条件。加快太原晋中公共服务一体化,重点推进医疗社保、汇兑结算、景点公交、通讯服务的互通互联;探索绿色出行方案,市城区启动电动公交及专用车道建设。

　　推进城乡共建。完成晋中城市总体规划修编,构建科学合理的城镇化布局。按照“一区两翼”总体布局,加快“大县城”试点和省级示范镇公共基础设施建设,发展特色小城镇和农村新社区。扎实推进城乡环境综合整治,强化城市市容和环境卫生管理,提高城市道路机械化清扫率,推进国家卫生县城、国家园林城市、省级园林县城创建工作。大力改善城乡交通条件,对市城区交通网络进行科学规划、改造提升,增设人行天桥,拓宽铁路桥下穿通道瓶颈,有效缓解交通拥堵问题;推动汾邢高速年内通车运营,开工建设319线左权县城段改线工程;加速推进集散公路、园区公路、通乡通村公路建设,新增通车里程200公里,完成400人以上自然村村通水泥(油)路100公里;晋中汽车客运总站建成投用;推进太焦铁路改造以及和邢铁路、阳泉大寨铁路建设。增强“水支撑”能力,做好东山调水、中部引黄5个受水县区域小水网建设,启动高校新区及城市西部退水改造工程,改善提高农村饮水安全标准。加快实施6项重点电网项目,建成220伏灵石两渡等11项输变电工程,推进新一轮农网升级改造。

　　创新建管机制。建立规范高效安全的城市建设投融资机制,鼓励社会资本参与城市公用设施投资运营。市城区探索城市建管分离、集中执法、数字城管、业务外包等新模式,建立“专家治市”的规划管理、规划实施、决策支持新机制。深化户籍制度改革,有序推进农业转移人口市民化,努力实现城镇基本公共服务常住人口全覆盖。

　　(六)坚持底线思维,突出生态文明,努力在改善环境质量上取得突破。

　　牢固树立“绿色、低碳、环保、健康”的发展理念,多措并举,综合施策,全面提升现代生态宜居水平,努力让晋中天更蓝、水更清、地更绿、环境更宜人。推进节能降耗。严格能评审查,推广合同能源管理,深入开展节能行动和能效对标。实施好新能源汽车试点及推广工作,加快推进电动公交车示范项目,力争年内建成一座电动汽车换电站,100辆电动公交车投入运营。大力发展绿色建筑,加快既有建筑节能改造。

　　狠抓减排治污。加快推进污染减排项目建设,扎实做好大气污染防治,全面改造市城区燃煤取暖锅炉,大力开展电力、水泥、焦化等行业脱硫脱硝和扬尘污染综合整治,坚决关停不达标企业;强化水污染防治,正阳污水二期工程年内建成投用,加快推进全市污水处理厂整合重组、管网扩建和中水回用。完善污染物排放许可制,建立生态修复多元化投入机制,试行第三方治理。实行最严格的环境保护监管制度,强化环保部门监管责任,加大环境违法案件查处力度。

　　加快生态建设。以创建省级林业生态市为抓手,重点加快平川现代林业展示区和东山生态经济林业示范区建设,集中打造108生态走廊,完成新造林合格面积50万亩以上,林木绿化率增加1.1个百分点。加大省级林业生态县创建力度,两年内实现全市通道两侧绿色和县城周边湿地、森林公园全覆盖。开发利用山庄窝铺,扶持发展生态庄园。

　　(七)坚持以人为本,突出体系建设,努力在构建和谐晋中上取得突破。

　　把改善民生作为第一责任,顺应广大群众的新向往、新追求、新期待,建机制,补短板,兜底线,切实解决群众后顾之忧,不断提升全市人民的幸福感。扎实做好就业和社会保障工作。引深省级创业型城市创建活动,建立政府投资和重大建设项目新增就业岗位预估、考核制度,整合市县两级人力资源市场,做好高校毕业生、农村转移劳动力、城镇困难人员、退役军人等重点群体就业保障工作,建立农民工工资保证金制度,推进创业孵化基地建设,转移农村劳动力3.87万人。要加快完善社会保障体系,建立统一的城乡居民基本养老保险制度,企业退休人员基本养老金提高10%;加快城乡居民大病保险和居民医保门诊统筹,逐步扩大我市异地就医联网结算范围,城镇居民基本医保和新农合人均财政补贴提高到320元;城乡低保标准每人每月分别提高到376元、203元;建立一级重度残疾人护理补贴和贫困一级残疾人生活补贴制度,每人每年补贴480元,对5000名贫困残疾人进行康复救助;落实好企业最低工资标准,制定出台我市机关事业单位工作人员带薪休假实施细则;提高农村五保供养水平,开展国家养老服务业综合改革试点工作,积极发展民办养老服务机构,完成市级公立老年养护院主体工程,全面开工建设晋中市殡仪馆;建立居民家庭经济状况核对机制,促进社会救助公平公正;完善社会救助和保障标准与物价上涨挂钩的联动机制,保持物价稳定;新开工保障性住房11559套,建成1.7万套,探索廉租房和公共租赁房保障对象并轨;继续加强住房公积金扩面征缴,确保公积金安全有效使用。

　　全面加快社会事业发展。引深国家级义务教育均衡发展试点,继续实施薄弱学校改造计划和学校标准化提档升级工程,启动市城区13所学校新改扩建工程,今年建成6所;实施第二轮学前教育三年行动计划,完成17所标准化公办幼儿园和35所农村幼儿园建设与改造任务;启动晋中职业技术学院新校区规划和筹建工作,改善职业高中办学条件。大力发展医药卫生事业,落实基本药物制度和基层医疗卫生机构运行新机制,推进县级公立医院改革并扩大试点范围,试点开展县域医疗卫生一体化综合改革。加快推进县级食品药品监管机构改革,全面推行生产经营者首负责任制、食品药品质量可追溯制和安全责任追究制。实施文化惠民工程和“全民文化行动”,加快“五馆一院”建设步伐,深化市直文艺院团实质性市场化改革。加快建设基本公共体育服务体系,继续打造城市社区500米健身圈工程,完成63个社区、100块场地建设任务,拓展体育公园场馆规模及设施功能。落实各级政府的文物保护责任,支持国防和军队建设,创建省级和全国双拥模范城,发展妇女儿童、老龄、档案和红十字会、慈善等事业,做好计划生育、民族、宗教、外事、侨务、对台、气象、史志、科普、地震等工作。

　　坚持不懈抓好安全生产。严格安全生产政府和企业两个主体责任,构建严密的责任落实机制。积极创建安全发展先进城市,全面落实党政同责新机制,强化安全准入制度,扎实推进安全生产标准化建设,动态实施安全生产分级分类管理,完善落实隐患举报奖励制度。按照“全覆盖、零容忍、严执法、重实效”要求,深入实施安全生产大检查、打非治违、专家会诊、危险化学品集中整治和汲取事故教训类比检查等行动,打牢安全生产基层基础。不断加大安全投入,强化人员培训,推进科技兴安,深化安全质量标准化建设。严格落实安全生产目标责任考核“一票否决制”,严肃查处各类生产安全事故,依法追究责任,坚决遏制重特大事故,努力减少一般事故,促进全市安全生产形势持续稳定好转。

　　加强和创新社会治理。扎实推进各类社会治理服务网格融合共建,进一步强化城镇社区网格化治理。切实加强信访稳定工作,建立完善畅通有序的诉求表达、心理干预、矛盾调处、权益保障机制,及时就地化解社会矛盾。强化防灾减灾能力和应急管理,妥善应对各类突发事件。深入推进平安晋中建设,加强社会治安综合治理,依法打击各种违法犯罪活动,维护国家安全,形成良好社会秩序,保障人民群众安居乐业。

　　广泛开展群众性精神文明创建活动。培育和践行社会主义核心价值观,大力加强社会公德、职业道德、家庭美德和个人品德教育,弘扬“晋中精神”,推进移风易俗,形成良好社会风尚。

　　(八)坚持为民服务,突出职能转变,努力在加强政府自身建设上取得突破。

　　始终牢记“打铁必须自身硬”,以开展党的群众路线教育实践活动为契机,按照为民务实清廉的要求,加快建设人民满意的服务型效能政府。强化依法行政。严格按照法定权限和程序行使权力、履行职责,自觉接受人大、政协和社会各界的监督。健全完善重大行政决策公众参与、专家论证、风险评估、合法性审查和集体讨论决定等制度,提高政府决策的科学化、民主化、法制化、规范化水平。加强行政复议,完善法律援助,推进政府法律顾问全覆盖。发展基层民主,做好第十届村委会换届选举工作。强化行政监察和审计监督,全面落实行政执法责任制和行政问责制,大力整治庸散懒等行政不作为和吃拿卡要等行政乱作为行为。深化政务公开,完善政府信息发布制度,让权力始终在阳光下运行。

　　推进高效施政。围绕审批事项少、办事效率高、运行成本低、行政流程透明目标,自我革命,简政放权,突出服务,切实转变政府职能。与专业机构合作对政府职能、结构、流程、制度进行全面改革优化,取消一切不增值行政环节。以晋中经济技术开发区、榆次工业园和祁县经济开发区为突破,大胆下放实权,深化行政审批改革,规范中介服务,实行项目投资负面清单和行政管理权力清单制度。推动政府购买公共服务改革,凡事务性管理服务原则上向社会购买,抓好政府购买服务的市场开发,培育发展相关服务产业。强化政府的市场监管职能,从注重事前审批向事中事后监管执法转变。建立完善以结果为导向的工作落实机制和常态化社会评议评价机制,不断提升政府的执行力和公信力。

　　狠抓廉洁从政。认真贯彻“八项规定”,严格执行“约法三章”和廉洁从政各项要求,努力做到严以修身、严以用权、严以律己,谋事要实、创业要实、做人要实。全面落实政府系统党风廉政建设责任制,严厉查处违纪违法案件,坚决纠正不正之风,深入推进反腐倡廉建设。大力弘扬艰苦奋斗优良传统,严格执行中央厉行节约、反对浪费的有关规定,严控“三公”经费,规范公务接待,真正把有限的公共财力用于推动发展和民生改善。加大“四风”整治力度,不断改进工作作风,坚持流水到头、落地有声,以铁石作风推动工作有效落实。

　　各位代表,民之所望、施政所向,政之所为、行之贵力。让我们在省委、省政府和市委的坚强领导下,紧紧依靠和团结带领全市人民,最大限度地凝聚改革发展的正能量,锐意进取,真抓实干,不断开创晋中转型跨越发展的新局面!
